# Unhealthy Lifestyle and Nutritional Habits Are Risk Factors for Cardiovascular Diseases Regardless of Professed Religion in University Students

**DOI:** 10.3390/ijerph15122872

**Published:** 2018-12-14

**Authors:** Silvia Navarro-Prado, Jacqueline Schmidt-RioValle, Miguel A. Montero-Alonso, Ángel Fernández-Aparicio, Emilio González-Jiménez

**Affiliations:** 1 Department of Nursing, Faculty of Health Sciences, University of Granada, 52071 Melilla, Spain; silnado@ugr.es (S.N.-P.); anfeapa@ugr.es (Á.F.-A.); 2 Department of Nursing, Faculty of Health Sciences, University of Granada, 18016 Granada, Spain; emigoji@ugr.es; 3 Department of Statistics and O.I. Faculty of Medicine, University of Granada, 18016 Granada, Spain; mmontero@ugr.es

**Keywords:** lifestyle, eating habits, cardiovascular disease, university students, religion

## Abstract

To date, few studies have evaluated the possible association between religion and nutritional habits, lifestyle and cardiovascular risk in the university population. This study identified differences in the eating habits of Christian and Muslim university students and determined a possible association between the impact of religion on their lifestyles and the parameters related to cardiovascular risk. A cross-sectional study was performed with a sample population of 257 students (22.4 ± 4.76 year) at the campus of the University of Granada in Melilla (Spain). An anthropometric evaluation and a dietary assessment were performed. Blood pressure was also measured. There was a higher prevalence of overweight (29.1%) among Christian university students. The prevalence of pre-hypertension was similar between Christians and Muslims (48.3%) but was higher among Christian males (74.5%). Christian students presented higher levels of visceral fat. Students of both religions ingested carbohydrates, saturated fatty acids and total cholesterol, proteins, sodium and alcohol in excess. Significant positive correlations were found between food energy, sweets, snacks, soft drinks and body mass index (BMI) in both sexes and between the consumption of sausages-fatty meats and the systolic blood pressure (SBP) and body adiposity index (BAI) variables. Muslim students were less likely to consume alcohol (odds ratio [OR] = 7.88, 95% confidence interval [CI] = 4.27, 14.54). Christian and Muslim students presented improvable lifestyles and intake patterns. The high intake of saturated fatty acids, total cholesterol, sodium and alcohol in Christian students could lead to the early development of cardiovascular disease.

## 1. Introduction

Recent studies in university students have demonstrated the existence of unhealthy lifestyles and unsuitable health behaviours [1,2,3]. According to VanKim et al. [4], since the university stage is a period of transition between adolescence and adulthood and is characterised by greater independence, autonomy and responsibility, it is often the first period in which young people make their own decisions about “what, how, where and when to eat.” It is thus a crucial phase for the adoption of long-lasting health habits and behaviours, including healthy eating patterns [5]. In this sense, other studies indicate unhealthy dietary practices in this group, such as rapid weight-loss diets, the omission of certain important food groups, consumption of high-calorie foods and foods with reduced nutritional value and the excessive intake of alcohol, among other toxins [6,7]. All of these behaviours can lead to deficiencies in macronutrients and micronutrients and other essential dietary components crucial for maintaining optimal nutritional health [8,9,10,11]. With all of these factors in mind, the acquisition and maintenance of nutritional practices and unhealthy lifestyles constitute an essential component in the aetiology of Chronic Noncommunicable Diseases (CNCDs), such as obesity and cardiovascular disease.

Various studies indicate that religion can act as a protective factor against the adoption of unhealthy nutritional habits and lifestyles among university populations [12]. Studies such as those published by Van der Meer Sanchez et al. [13] and Gomes et al. [14] in a Brazilian university population suggest that religion can positively influence the adoption of healthy lifestyles and can act as a protective factor against the consumption of drugs and alcohol in this group. Similar results were obtained by El Ansari et al. [15], who studied a population of 3220 students of different religions from seven UK universities and found that religion was a protective factor against excessive alcohol consumption. The authors conclude that fostering the spiritual health of students could have a preventive role against the alcohol consumption as well as other harmful habits in the university environment. Along this same line, Neighbors et al. [16], in their study of 1124 American university students, found that religious faith was negatively associated with alcohol consumption; students who placed greater importance on religion tended to consume less alcohol despite living in an environment where drinking was a normative behaviour.

However, to date, few studies have focused on the relationship between the nutritional patterns and lifestyles of university students according to their religion and the health parameters related to cardiovascular risk [17]. Sørensen et al. [18] studied a sample population of 120,000 young Norwegian adults and found a positive association between religion and blood pressure levels. In other words, those young people of both sexes who self-identified as Christian or practicing Muslims had lower blood pressure levels. This suggests that religion could act as a protective factor against cardiovascular risk in both sexes. The authors conclude that there is a need to carry out new studies to analyse the behaviour of these variables in different geographical contexts. In this regard, Melilla, a Spanish university city located in North Africa with large Christian and Muslim university populations, is the ideal setting for evaluating whether this religious plurality is associated with nutritional habits and lifestyles of university students and the possible relationships of these factors to cardiovascular risk [19]. The main objectives of this study were to characterise the eating habits and lifestyles of the university students of the Melilla campus and to detect a possible association between religion and lifestyles and the parameters related to cardiovascular risk.

## 2. Materials and Methods

### 2.1. Study Design and Sampling

This is a cross-sectional study carried out during the 2013–2014 academic year, in which 257 university students participated (21.63%), 141 of the Christian faith and 116 of the Muslim faith, 22.4 ± 4.76 years of age. All of the students were selected by random sampling (*n* = 1188 students) from the total population of the university campus of the city of Melilla, a Spanish enclave located on the northwest coast of Africa, across the Alboran Sea from the Spanish provinces of Granada and Almeria. It is characterized by its rich cultural traditions, fruit of the coexistence of different ethnic groups and cultures over the centuries. This is reflected in its large university population of Christian and Muslim students. Despite its location, Melilla is a modern, westernized city.

### 2.2. Data Collection

**Figure 1:**
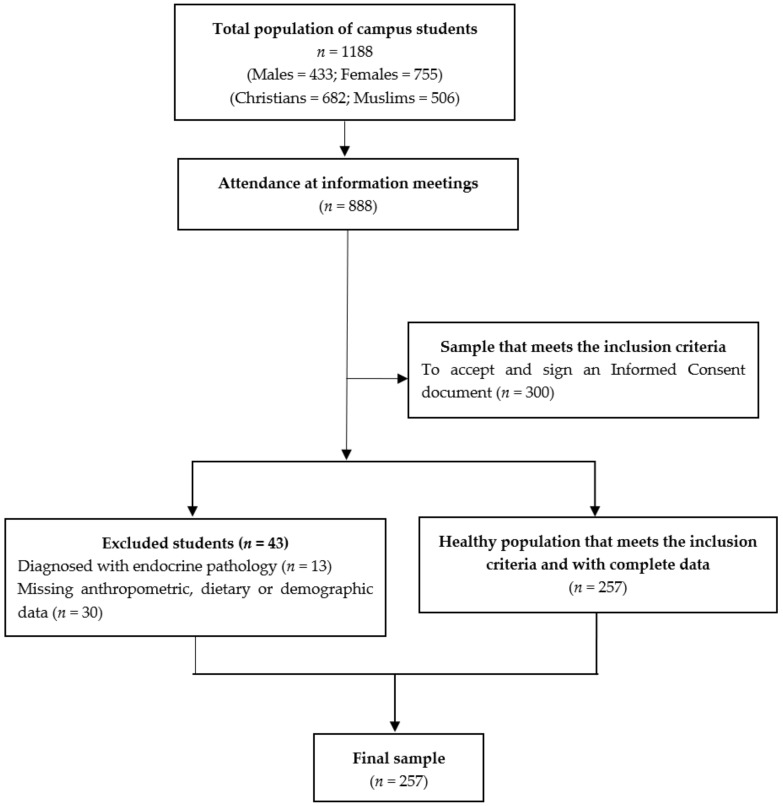
Flow chart of the recruitment progress.

One of the inclusion criteria for the study was to be enrolled in a Degree Program offered by one of the three university centres that make up the Melilla campus, namely, the Faculty of Educational and Sport Sciences, Faculty of Social Sciences and Law and Faculty of Health Sciences. Another essential criterion was to agree and sign an Informed Consent document. Students were excluded from the study if they had been diagnosed with endocrine and metabolic pathologies or if they were not willing to participate in the study. The flow chart (Figure 1) summarises the process of selecting the participants.

During the month of September 2013, on the Melilla university campus, information meetings were held for all campus students (*n* = 1188). However, only 888 students from the total population attended all the meetings. In these meetings, the attendees were informed about the different assessments and the questionnaires that they would have to complete if they ultimately decided to participate in the study. All attendees received an informed consent form, which included a detailed description of the study. At these meetings, screening for inclusion criteria was completed and a total of 300 students were selected. Of these students, 43 were excluded for different reasons (namely, being diagnosed with an endocrine pathology (*n* = 13) and incomplete anthropometric, dietary, or demographic data (*n* = 30)). The final sample included 257 students, all of whom were healthy subjects who met the inclusion criteria and had provided complete data. The full evaluations of the 257 participants were conducted in October 2013 and comprised an anthropometric assessment, an analysis of body composition and a dietary assessment.

The study was approved by the Ministry of Education and Youth of the Government of Melilla. Furthermore, the study, including its model of informed consent, was approved by the Ethics Committee of the University of Granada (Code 841). All of the participants gave their authorisation in writing and the data were coded to guarantee confidentiality. This research was performed in strict compliance with the international code of medical ethics established by the World Medical Association and the Declaration of Helsinki.

### 2.3. Dietary Assessment, Determination of Nutritional Adequacy, Lifestyle and Sociodemographic Variables

A dietary assessment was conducted using an ad hoc questionnaire about the frequency of self-administered food consumption. This instrument was an adapted version of a questionnaire (lifestyle and sociodemographic data, eating habits and food consumption), which was an elaboration of an instrument previously validated by González et al. [20]. The ad hoc questionnaire contained a nutrition section that compiled information about all food groups, including foods characteristic of the Muslim religion. Furthermore, a 72-h diet recall interview, considering intakes on Thursday, Friday and Saturday, was used to capture weekly variations on weekdays and the weekend. In a face-to-face interview with trained investigators, individuals were asked to recall all food consumed in the preceding 72 h, including beverages. Standard household measures and pictorial food models were employed during the interviews to define amounts when requested. Completed food records were analysed using a computerised nutrient analysis program (Nutriber 1.1.5). The adequacy of each nutrient was determined by comparing nutrient levels with each dietary reference value in the Spanish Dietary Reference Intakes (DRIs) [21].

The lifestyle and sociodemographic data were evaluated from an additional section of the same questionnaire used in the nutritional assessment. This section collected the following variables: person in charge of the daily menu (yourself/other), regular daily meals (breakfast, mid-morning snack, lunch, mid-afternoon snack, dinner), nibbling between meals, use of “miracle” slimming diets, consumption of weight-loss products, alcohol, cigarette consumption and use of illegal drugs. The sociodemographic variables assessed included the average age of the father and mother, the education levels of both parents (no education, primary or secondary school, vocational studies or *Bachillerato* [upper secondary] and university) and the professional occupations of the father (hospitality/service sector, merchant, or civil servant) and mother (hospitality/service sector, housewife, or “healthcare” civil servant). Information regarding paternal and/or maternal obesity status was also collected. This was done by requiring participants to present a medical certificate stating the presence or absence of obesity in their parents. This section also included a variable regarding self-identified religion, in which the student declared the religion he/she professed and practised (Christianity or Islam). This variable was adapted from the Religious Attitudes Questionnaire (*Cuestionario de Actitudes Religiosas—CAR in Spanish*), developed and validated by Elzo [22].

### 2.4. Anthropometric Evaluation and Body Composition

The anthropometric assessment was carried out individually and with sufficient privacy in a classroom provided by the Faculty of Health Sciences. The anthropometric variables, including the waist-to-hip ratio (WHR), were measured following the recommendations of the International Society for the Advancement of Kinanthropometry [23], the first thing in the morning, between 7:00 and 10:00 a.m. and after an overnight fast. Body weight (kg) was measured with the subjects in their underwear and without shoes, using an electronic scale (Tanita BC-418MA^®^, Hamburg, Germany) with a low technical error of measurement (technical measurement error [TEM] = 0.510%). Height (cm) was measured using a stadiometer (Seca^®^ 274, Hamburg, Germany, TEM = 0.01%). The body mass index (BMI) was calculated by dividing the weight in kilograms, by the height squared (kg/m^2^). The BMIs of the subjects were grouped in accordance with the categories established by the World Health Organisation [24]. The study of body composition was performed with an electronic body composition analyser (Tanita BC-418MA^®^, Hamburg, Germany), which is one of the most frequently recommended methods for estimating body composition because of its low cost, technical simplicity and its non-invasive nature [25]. Our study used this electronic scale to analyse fat mass, lean mass, muscle mass, total water, bone mass, basal metabolism and visceral fat. All measurements were taken by the same trained researcher.

### 2.5. Blood Pressure

Blood pressure (BP) was measured with a DinaMap vital signs monitor (model BP 8800, Critikon, Inc., Tampa, FL, USA, TEM = 0.598). To measure resting BP, subjects sat in a semi-reclined position with arms relaxed, supported and with the midpoint of the arm at the level of the heart. After a resting period of at least 5 min, BP was measured twice and was categorised according to BP tables from the European Society of Hypertension (ESH)/European Society of Cardiology (ESC) in adults [26], with normotension defined as systolic (120–129 mmHg) and diastolic (80–84 mmHg). Pre-hypertension was defined as systolic (130–139 mmHg) and diastolic (85–89 mmHg) and hypertension was defined as systolic (≥140–159 mmHg) and diastolic (≥90–99 mmHg).

### 2.6. Statistical Analysis

The statistical analysis was performed with IBM SPSS 20 software (SPSS Inc., Chicago, IL, USA). Means and standard deviations were the descriptive statistics for the quantitative variables. Qualitative variables were described in terms of proportions. Pairs of means were compared using two-sample Student’s t-tests (with Levene’s test for equality of variances) for normally distributed variables and the Wilcoxon test for non-normally distributed variables. In addition, the chi-square test was employed for comparing categorical variables. Correlations between the variables were assessed using Pearson’s correlation coefficient. Odds ratios were calculated using binary logistic regression analysis with religion as the dependent variable (95% confidence intervals, CI). The first step included models that assessed the relationship between each determinant and overweight/obesity and the corresponding odds ratios adjusted for the following variables: normal weight, WHR, visceral fat indicator, hypertension, fat mass (%), cigarette consumption, consumption of alcoholic beverages and practising sports. In the second step, odds ratios were adjusted by religion and sex. The normality of the distributions was verified by using the Kolmogorov-Smirnov test. The level of significance was *p* < 0.05.

## 3. Results

### 3.1. Parental Sociodemographic Characteristics

The sample population consisted of 141 Christian students (51 males and 90 females) and 116 Muslim students (37 males and 79 females). Regarding the education levels and professional occupations of the fathers and mothers, there were statistically significant differences (*p* < 0.001) between the parents of Christian and Muslim students. These results are shown more clearly in Table 1. Regarding the obesity status of the parents, statistically significant differences were observed (*p* < 0.001) for the fathers. Thus, up to 17.0% of the fathers of the Christian students suffered from obesity, compared with 6.0% of the fathers of the Muslim students.

### 3.2. Anthropometric Characteristics, Bone Mass, Blood Pressure Value and Lifestyle Characteristics

As shown in Table 2, there was a higher prevalence of normal weight (75.9%) among the Muslim students, followed by a higher prevalence of overweight (29.1%) among the university students of the Christian faith, with a greater proportion among males. In terms of blood pressure levels, the average prevalence of subjects with pre-hypertension was similar between the Christian and Muslim students (48.3%), although the prevalence of pre-hypertension was higher among males, particularly among the Christian students (74.5%). Visceral fat levels were higher among Christian males and females (2.73), with the highest values (3.75) found among Christian males. Regarding the person responsible for preparing the daily menu, statistically significant differences were observed (*p* < 0.001) between the Christian and Muslim students; 46.8% of the Christian students were responsible for preparing their own menu each day, compared with 16.4% of the Muslim students.

With respect to regular daily meals, there were significant differences (*p* < 0.001), depending on religion, for breakfast. More specifically, 80.9% of the Christian students ate breakfast daily in comparison to 61.2% of the Muslim students. Furthermore, 92.2% of Christian students ate an evening meal versus 68.1% among Muslims. As far as nibbling between meals, statistically significant differences were observed (*p* < 0.001) between the Christian and Muslim students, with this practice being higher among the Muslims (64.7%), particularly among females (65.8%).

Concerning the consumption of weight-loss products, statistically significant differences (*p* < 0.001) were also observed between both groups. These products were more frequently consumed by the Christian students (27%), mainly by females (28.9%). Regarding the consumption of alcoholic beverages, there were also statistically significant differences (*p* < 0.001) between the Christians and Muslims, with consumption being much higher among the Christian students (79.4%) compared to the Muslims (28.4%). In terms of the consumption of illegal drugs, no statistically significant differences were observed between the Christian and Muslim students. However, their consumption was higher among the Muslims (10.3%), particularly among males (18.9%).

### 3.3. Daily Intake of Energy, Macronutrients and Micronutrients

The results showed a high carbohydrate intake among males, with statistically significant differences (*p* < 0.001), between the Christians and Muslims (Table 3). Regarding the intake of lipids, even though there were no statistically significant differences between Christians and Muslims, all of the males and females exceeded intake recommendations. There were also statistically significant differences (*p* < 0.001) in protein intake between the Christian and Muslim students, with a very high average intake among Christian males, which did not correspond to intake recommendations. Statistically significant differences (*p* < 0.001) in sodium intake were observed between the Christian and Muslim students, with the highest intake among Christian males, 25.5% of whom exceeded intake recommendations. Concerning the intake of saturated fatty acids (SFAs), even though there were no statistically significant differences between the Christian and Muslim students, there were high average intake levels among males of both groups. None of the Christian males (100%) complied with intake recommendations, followed by 97.3% of Muslim males. Total cholesterol intake was above the recommended level, particularly among males in both groups, although there were no statistically significant differences between the Christian and Muslim students. Regarding alcohol intake, statistically significant differences (*p* < 0.001) were observed between the Christian and Muslim students, with alcohol consumption being considerably higher among Christian males and females.

### 3.4. Correlations (R) between Energy and Macronutrient Intake, Food Group Serving Intake, Body Mass Index, Blood Pressure, Waist-To-Hip Ratio and Body Adiposity Index

Table 4 shows the correlations between the intake of the principal food groups and parameters related to cardiovascular risk in males and females of the Christian faith. Significant positive correlations were observed between the food energy variable and the body mass index (BMI) and body adiposity index (BAI) variables in males (*p* < 0.01). There was also a significant positive correlation between the diastolic blood pressure and the intake of bread, grains and pasta among males (*p* < 0.05). Negative correlations were observed in both genders between the consumption of vegetables, fruit, legumes, fish and seafood and BMI, BP (systolic blood pressure–SBP and diastolic blood pressure–DBP), WHR and BAI. Likewise, among males, there was a significant negative correlation between the intake of lean meats and the WHR (*p* < 0.05). The results also showed positive correlations between the intake of eggs, sausages and fatty meats and the BMI, SBP and BAI variables among males. In addition, positive correlations were observed between the intake of sweets, snacks and soft drinks and the SBP and BAI variables; their intake also correlated strongly and very significantly with the BMI. Significant positive correlations were found between the food energy and the consumption of sweets, snacks and soft drinks variable and BMI in females and between the ingestion of sausages and fatty meats and the BAI.

Table 5 also shows correlations between the intake of food groups and the same parameters linked to cardiovascular risk in students of the Muslim faith. Significant positive correlations were observed between food energy and the BMI and WHR variables in both genders (*p* < 0.05). There were also strong correlations, although not significant, in both sexes with the BAI variable. In males, significant negative correlations were observed between the consumption of greens and vegetables and the BMI and BAI (*p* < 0.05) and for the consumption of legumes and SBP (*p* < 0.01) and fish and seafood and SBP (*p* < 0.05). In males, significant negative correlations (*p* < 0.01) were also observed between the consumption of lean meats and the BMI and BAI variables. Among females, these correlations were not significant and occurred only with the BAI variable. In males, positive correlations were also found between the intake of sausages and fatty meats and the BMI, SBP and BAI, with statistically significant differences in the latter (*p* < 0.05). In females, the intake of fatty meats and sausages correlated positively and significantly (*p* < 0.05) with the BMI, SBP and BAI. The consumption of sweets, snacks and soft drinks correlated strongly and positively with the BMI and BAI variables in both genders, although more significantly with males (*p* < 0.01).

### 3.5. Influence of Belonging to the Christian or Muslim Religion on Variables Related to Cardiovascular Risk

There was no association between belonging to the Christian or Muslim religions and maintaining an adequate nutritional status (normal weight), having a normal score on the WHR, normal blood pressure levels and fat mass (%) and practising sports (see Table 6). However, the number of students with normal levels of visceral fat indicator compared to those with high levels was 3.83 (95% confidence interval [CI] = 1.06), 13.77 times higher in Christian students than in Muslim students. The same was true for cigarette consumption (OR = 2.08, 95% CI = 1.15, 3.88) and consumption of alcoholic beverages (OR = 7.88, 95% CI = 4.27, 14.54).

## 4. Discussion

The results obtained in this study showed differences in sociodemographics, dietary habits and lifestyle among the Christian and Muslim students in Melilla. It was observed that both the fathers and mothers of the Christian students had higher education levels. The mothers of the Christian students worked mostly outside the home, unlike their Muslim counterparts, who were mostly housewives. These and other aspects could modulate the habits and lifestyles of their children [27], particularly among the Christian students during the university stage, a critical period for increased independence from the family nucleus [28,29]. Regarding the parents’ obesity status, the results showed that the fathers of the Christian students had a higher prevalence of obesity. According to Whitaker et al. [30] regardless of genetic factors, parents and children share the same socioecological environment. Consequently, paternal obesity could be a significant factor that conditions their children’s habits and customs during the university stage.

Regarding the nutritional status of the students, there was a higher prevalence of normal weight among the Muslim students, followed by a higher percentage of overweight among the Christians (29.1%), especially among males. These results differ from those obtained by Abdel-Megeid et al. [31], who reported an overweight rate of 21% in a population of 312 Saudi university students, also with a higher prevalence among the male students (23%). Other studies, such as Yahia et al. [32] of a population of 220 Muslim students from the Lebanese American University, found an overweight prevalence of 37.5% among males compared with 12.5% among females, indicating the need to implement programmes of nutritional health among this social group. Regarding blood pressure levels, the mean prevalence rates of pre-hypertension were similar between the Christian and Muslim students (48.3%). According to sex, pre-hypertension was much higher among Christian males (74.5%).

These results contrasted with 22% for pre-hypertension observed by Abdel-Megeid et al. [31], among Saudi university students of both sexes. In terms of visceral fat, the results revealed a higher visceral fat deposit among the Christian males and females, particularly among Christian males. In this regard, Al-Rethaiaa et al. [33] described very low visceral fat levels in a population of 357 Saudi Muslim university students. According to Marques et al. [34], this circumstance is relevant if we consider that visceral fat is closely related to the development of cardiovascular pathology.

Concerning the person responsible for preparing the daily menu, 46.8% of the Christian students indicated that they were responsible for preparing their own menu, compared with 16.4% of the Muslim students. These data could explain the higher prevalence of overweight students among Christians compared with Muslims, whose mothers prepared the family menu every day. Additionally, the results showed differences in the regularity of daily meals, with more Muslim students skipping breakfast (38.8%) and the evening meal (31.9%). Studies of university populations from different countries show variations in the daily partaking of breakfast, with 80% in the United States [35], 90% in France [36] and 72.5% in Australia [37]. However, in our study, the daily practice of eating breakfast was more frequent than the result obtained by Yahia et al. [32] at the University of Lebanon (52.7%). In any case, these results should lead to the design and implementation of educational interventions for making college students aware of the importance of not skipping any of the main daily meals, especially breakfast.

Regarding nibbling between meals, the results showed significant differences between Christians and Muslims. Our study showed that nibbling was more frequent among the Muslim students, especially female Muslims. According to Oliveras et al. [38], regular nibbling between the three daily meals can be a significant source of calorie intake because the ingested foods are frequently rich in fat and sodium.

As for the consumption of weight-loss products, there were also significant differences between both groups. This practice was more frequent among Christian students, particularly among females (28.9%), which coincides with previous studies of Colombian and Belgian university students [39,40]. According to Bonfanti et al. [41], this practice can lead to nutritional deficiencies, hormonal disorders, slowing of the basal metabolism, and, consequently, the dreaded rebound effect, which causes the person to rapidly regain the weight lost.

Regarding the consumption of alcohol, significant differences were found between Christians and Muslims, with a much higher consumption among the Christian students (79.4%). According to Isralowitz et al. [42], this finding could be explained by the protective effect that certain religions, such as Islam, have on toxic consumption, with an explicit prohibition of alcohol consumption. However, Al-Ansari et al. [43] reported a significant increase in its consumption, suggesting the need to review the restrictive alcohol policies in predominantly Muslim countries.

In our study, we observed significant differences between Christian and Muslim students in the intake of carbohydrates and proteins, with a higher intake especially among the Christian males, which was not in consonance with the intake recommendations for the Spanish population. These results partially coincide with those of Montero et al. [44], who reported a deficient intake of carbohydrates but an excess of fats and proteins among university students in Madrid. De Piero et al. [45] reported an excessive intake of carbohydrates and proteins to the detriment of lipids among a population of Argentinian university students, which coincided with the results obtained in our study.

Significant differences in sodium intake were found between the Christian and Muslim students, with a higher intake among Christian males, which matched data described by Perez-Garcia et al. [46] for university students of Valladolid. The intake of Vitamin B9 or folic acid was very low in all cases, a worrisome situation because of its importance for homocysteine metabolism, whose elevated plasma levels are considered to be an independent risk factor for cardiovascular disease [47]. Regarding the intake of saturated fatty acids (SFAs) and total cholesterol, even though there were no significant differences between Christians and Muslims, the males of both groups exceeded intake recommendations for the Spanish population, similar to what was reported for other university populations in Spain [48] and Colombia [49]. This situation is very disturbing considering that the accumulation of cholesterol in the blood and its deposition on the vascular endothelium favours the formation of atheromatous plaques and inflammation, processes conducive to atherosclerotic disease. Concerning alcohol intake, while its consumption was still high among Christian males and females, it was within established recommended limits (<30 g/day). These results contrast with those described by Da Silva et al. [50], who reported alcohol intake levels well above what is recommended among Brazilian university students, regardless of religion.

Our results showed positive correlations between: (i) the intake of food energy; (ii) certain food groups such as sausage, fatty meats, sweets, snacks and soft drinks; and (iii) the parameters predictive of cardiovascular risk, such as BMI, WHR, or BAI. The results also showed negative correlations between the consumption of greens and vegetables and parameters, such as BMI and BAI and between the consumption of legumes, fish and seafood and SBP. Similar results were obtained in previous studies that described positive correlations between the frequent intake of hypercaloric foods, salty snacks and foods rich in saturated fats and the BMI, WHR, BAI, or SBP values. The results obtained are relevant if we consider that a diet rich in saturated fats can increase serum levels of low-density lipoprotein cholesterol (LDL-C), favouring the early development of atheromatous plaques [51]. In addition, a predominantly fatty diet can induce and favour the process of arterial endothelial inflammation, further increasing the risk of heart disease and hypertension at an early age [52].

There was no influence between belonging to the Christian or Muslim religions and presenting normal values of body weight, WHR, blood pressure and fat mass (%) or practising sports. According to Ruíz Laso [53], this circumstance could be explained by the globalising effects of trends and social patterns in these young people, which could influence their decisions. The data, both raw and adjusted by sex, showed that the Muslim students had a lower risk of having high levels of visceral fat, which could be associated with their lower prevalence of overweight. Regarding tobacco consumption, the raw data indicate that there was also a lower risk of smoking among Muslim students, a fact that cannot be justified only by religious prohibitions since its consumption is only prohibited during the month of Ramadan [54]. However, according to Wang et al. [55], family pressure and the influence of tradition could justify this tendency towards a lower smoking habit. In addition, with the raw data and adjusting for sex, it was observed that the Muslim students were less likely to consume alcohol, a fact that could be explained by the Islamic prohibition on the consumption of alcoholic beverages [54].

This study has some strengths and limitations. Among the strengths is its pioneering nature, since it analysed the possible influence of religion on nutritional habits, lifestyle and cardiovascular risk among university students. Among its limitations is the cross-sectional nature of the study, which makes it impossible to establish causality, or to collect blood samples for biochemical studies. In addition, the use of food frequency questionnaires is another possible limitation because of the difficulties of performing a food recall in the university population. Another relevant limitation was the small size of the sample analysed, thus making it impossible extrapolate the results to other populations. Consequently, the results should be interpreted with caution. However, we believe that these limitations do not invalidate the results obtained.

## 5. Conclusions

The nutritional and health habits of university students in Melilla should be improved since the intake of carbohydrates, proteins, saturated fats and total cholesterol of Christian and Muslim students is not in consonance with the intake recommendations for the Spanish population. All of these factors, along with a high intake of sodium, could lead to the early development of cardiovascular disease. Belonging to either the Christian or Muslim religions did not influence normal body weight, WHR, blood pressure and fat mass (%) or the tendency to practise sports, although Muslim students were found to be at a lower risk of high levels of visceral fat, smoking and alcohol consumption. Therefore, there is an urgent need to implement comprehensive intervention studies adapted to each religion to analyse the factors that determine the lifestyle and eating habits of university students, with the particular goal of preventing future health problems and cardiovascular disease. It would be useful to design and implement a specific intervention program adapted to each religion in order to raise awareness and expand knowledge about healthy eating habits and lifestyles with a view to promoting self-care among the Melilla university population.

## Figures and Tables

**Table 1:** Parental socioeconomic characteristics of Christian and Muslims students.

Variables	Christian (*n* = 141)	Muslim (*n* = 116)
Mean age of the mother	51.84 ± 6.886	49.18 ± 7.455
Mean age of the father	54.54 ± 7.152	54.43 ± 7.873
Education of the father *		
No education	0 (0%)	7 (6.0%)
Primary or secondary school	54 (38.3%)	70 (60.3%)
Vocational studies or *Bachillerato*	47 (33.3%)	25 (21.6%)
University	40 (28.4%)	14 (12.1%)
Education of the mother *		
No education	1 (0.7%)	2 (1.7%)
Primary or secondary school	58 (38.3%)	48 (67.2%)
Vocational studies or *Bachillerato*	49 (34.8%)	18 (15.5%)
University	37 (26.2%)	18 (15.5%)
Father’s occupation *		
Hospitality/Services sector	49 (54.8%)	59 (50.9%)
Merchant	30 (21.3%)	45 (38.8%)
Civil servant	62 (44.0%)	12 (10.3%)
Mother’s occupation *		
Hospitality/Services sector	27 (19.1%)	16 (13.8%)
Housewife	49 (34.8%)	82 (70.7%)
Civil servant (healthcare)	65 (46.1%)	18 (15.5%)
Father’s obesity *		
Yes	24 (17.0%)	7 (6.0%)
No	117 (83.0%)	109 (94.0%)
Mother’s obesity		
Yes	19 (13.5%)	16 (13.8%)
No	122 (86.5%)	100 (86.2%)

Data are mean values ± SDs and percentages. * Chi-square test according to Christian or Muslim religion; *p* < 0.001.

**Table 2:** Anthropometric characteristics, bone mass, blood pressure value and lifestyle characteristics among Christian and Muslim males and females participating in the study.

Variables	Christian (*n* = 141)		Muslim (*n* = 116)		Total Christian (*n* = 141)	Total Muslim (*n* = 116)
	Males (*n* = 51)	Females (*n* = 90)	Males (*n* = 37)	Females (*n* = 79)		
Mean age (year)	22.27 ± 4.35	23.30 ± 6.01	20.97 ± 2.72	21.42 ± 3.90	22.93 ± 5.47	21.28 ± 3.56
Height (cm)	178.50 ± 7.12	163.77 ± 5.74	178.24 ± 7.15	165.67 ± 6.04	169.097 ± 9.46	169.68 ± 8.68
Weight (kg)	80.32 ± 12.52	60.46 ± 9.85	75.35 ± 12.04	62.10 ± 10.50	67.64 ± 14.46	66.32 ± 12.59
BMI (kg/m^2^)	25.19 ± 3.56	22.54 ± 3.45	23.70 ± 3.28	22.60 ± 3.53	23.50 ± 3.70	22.95 ± 3.48
Weight class						
Normal weight	29 (56.9%)	71 (78.9%)	23 (62.2%)	65 (82.3%)	100 (70.9%)	88 (75.9%)
Overweight	22 (43.1%)	19 (21.1%)	14 (37.8%)	14 (17.7%)	41 (29.1%)	28 (24.1%)
Waist circumference (cm)	85.44 ± 10.14	74.06 ± 8.34	82.95 ± 8.13	75.23 ± 8.96	78.18 ± 10.54	77.69 ± 9.39
Hip circumference (cm)	104.65 ± 12.63	100.16 ± 8.62	102.08 ± 8.00	101.42 ± 8.22	101.78 ± 10.43	101.63 ± 8.13
WHR	0.82 ± 0.09	0.74 ± 0.08	0.81 ± 0.05	0.74 ± 0.07	0.77 ± 0.09	0.76 ± 0.07
BAI (cm/m^1.5–18^)	25.92 ± 5.21	29.86 ± 4.44	24.99 ± 3.84	29.63 ± 4.15	28.44 ± 5.08	28.15 ± 4.58
ABSI (cm/(kg/m^2^)0.66/cm^0.5^)	0.076 ± 0.00	0.074 ± 0.00	0.077 ± 0.00	0.075 ± 0.00	0.0751 ± 0.006	0.0757 ± 0.005
SBP (mmHg)	121.42 ± 14.29	111.62 ± 11.35	123.11 ± 11.00	112.95 ± 11.32	115.17 ± 13.31	116.19 ± 12.14
DBP (mmHg)	70.22 ± 13.20	65.34 ± 7.904	72.05 ± 7.19	67.18 ± 6.29	66.75 ± 10.84	68.73 ± 6.95
Normal blood pressure	10 (19.6%)	60 (66.7%)	10 (27.0%)	48 (60.7%)	70 (49.6%)	58 (50.0%)
Pre-hypertensive	38 (74.5%)	30 (33.3%)	26 (70.3%)	30 (38.0%)	68 (48.3%)	56 (48.3%)
Hypertensive	3 (5.9%)	0	1 (2.7%)	1 (1.3%)	3 (2.1%)	2 (1.7%)
Fat mass (kg)	16.99 ± 11.12	16.97 ± 8.12	13.05 ± 6.26	17.60 ± 7.93	16.97 ± 9.28	16.15 ± 7.71
Fat mass (%)	19.17 ± 9.82	25.96 ± 7.44	16.72 ± 6.77	26.47 ± 7.31	23.51 ± 8.97	23.36 ± 8.45
Lean mass (kg)	64.57 ± 7.61	44.17 ± 3.85	62.65 ± 8.18	44.84 ± 4.25	51.55 ± 11.26	50.52 ± 10.13
Muscle mass (kg)	61.91 ± 6.60	42.01 ± 3.44	59.80 ± 7.92	42.54 ± 4.27	49.20 ± 10.73	48.04 ± 9.86
Bone mass (kg)	3.24 ± 0.33	2.27 ± 0.16	3.13 ± 0.38	2.27 ± 0.21	2.62 ± 0.52	2.55 ± 0.48
BUA left calcaneus (dB/MHz)	105.76 ± 19.37	86.22 ± 17.17	107.62 ± 20.25	89.80 ± 16.07	93.29 ± 20.25	95.48 ± 19.31
BUA right calcaneus (dB/MHz)	102.78 ± 17.10	84.76 ± 17.28	102.86 ± 15.57	87.10 ± 16.15	91.28 ± 19.23	92.13 ± 17.53
Basal metabolism (kcal/day)	1931.86 ± 244.92	1380.09 ± 111.95	1837.14 ± 311.66	1403.05 ± 136.74	1579.67 ± 316.51	1541.51 ± 290.47
Visceral fat (represented as levels) *	3.75 ± 2.82	2.15 ± 2.02	2.73 ± 1.75	2.04 ± 1.68	2.73 ± 2.45	2.26 ± 1.72
Person in charge of daily menu *						
Yourself	22 (43.1%)	44 (48.9%)	1 (2.7%)	18 (22.8%)	66 (46.8%)	19 (16.4%)
Other (mother)	29 (56.9%)	46 (51.1%)	36 (97.3%)	61 (77.2%)	75 (53.2%)	97 (83.6%)
Regular daily meals						
Breakfast (Yes) *	39 (76.5%)	75 (83.3%)	21 (56.8%)	50 (66.3%)	114 (80.9%)	71 (61.2%)
Mid-morning snack (Yes)	20 (39.2%)	27 (30.0%)	14 (37.8%)	23 (29.1%)	47 (33.3%)	37 (31.9%)
Lunch (Yes)	50 (98.0%)	90 (100%)	34 (91.9%)	77 (97.5%)	140 (99.3%)	111 (95.7%)
Mid-afternoon snack (Yes)	30 (58.8%)	38 (42.2%)	18 (48.6%)	33 (41.8%)	68 (48.2%)	51 (44%)
Dinner (Yes) *	46 (90.2%)	84 (93.3%)	30 (81.1%)	49 (62.0%)	130 (92.2%)	79 (68.1%)
Nibbling between meals (Yes) *	25 (49.0%)	49 (54.4%)	23 (62.2%)	52 (65.8%)	74 (52.5%)	75 (64.7%)
Use of “miracle” slimming diets (Yes)	7 (13.7%)	21 (23.3%)	3 (8.1%)	13 (16.5%)	28 (19.9%)	16 (13.8%)
Consumption of weight loss products (Yes) *	12 (23.5%)	26 (28.9%)	4 (10.8%)	7 (8.9%)	38 (27%)	11 (9.5%)
Consumption of alcoholic beverages (Yes) *	43 (84.3%)	69 (76.7%)	10 (27.0%)	23 (29.1%)	112 (79.4%)	33 (28.4%)
Cigarette consumption (Yes)	14 (27.5%)	25 (27.8%)	7 (18.9%)	11 (13.9%)	39 (27.7%)	18 (15.5%)
Use of illegal drugs (Yes)	3 (5.9%)	4 (4.4%)	7 (18.9%)	5 (6.3%)	7 (5%)	12 (10.3%)

Data are mean values ± SDs and percentages. Abbreviations: BMI: Body mass index, WHR: Waist-to-hip ratio, BAI: Body adiposity index, ABSI: A Body Shape Index, SBP: Systolic blood pressure, DBP: Diastolic blood pressure, BUA: Broadband ultrasound attenuation coefficient. Normal blood pressure: Systolic (120–129 mmHg) and Diastolic (80–84 mmHg); Pre-Hypertensive: Systolic (130–139 mmHg) and Diastolic (85–89 mmHg); Hypertensive: Systolic (≥140–159 mmHg) and Diastolic (≥90–99 mmHg); Visceral fat levels: Below 9 = Standard; 10–14 = High; Above 15 = Very high. * Chi-square test according to Christian or Muslim religion; *p* < 0.001.

**Table 3:** Daily intake of energy, macronutrients and micronutrients among Christian and Muslim males and females participating in the study.

	Christian (*n* = 141)				Muslim (*n* = 116)			
	Males (*n* = 51)		Females (*n* = 90)		Males (*n* = 37)		Females (*n* = 79)	
	Mean ± SD	Not-Meeting DRIs (%)	Mean ± SD	Not-Meeting DRIs (%)	Mean ± SD	Not-Meeting DRIs (%)	Mean ± SD	Not-Meeting DRIs (%)
Energy (kcal)	2294.38 ± 665.25	30 (58.8%)	1920.36 ± 545.21	59 (65.6%)	2217.37 ± 699.46	16 (43.2%)	2008.67 ± 690.79	31 (39.2%)
Carbohydrates (g)	250.61 ± 93.87 **	48 (94.1%)	211.93 ± 67.43 **	82 (91.1%)	265.22 ± 91.48	34 (91.9%)	242.83 ± 95.94	73 (92.4%)
Total fat (g)	92.80 ± 31.43	51 (100%)	81.38 ± 28.12	90 (100%)	87.73 ± 31.79	37 (100%)	81.90 ± 31.45	79 (100%)
Proteins (g)	105.77 ± 37.62 **	51 (100%)	79.45 ± 18.81 **	86 (95.6%)	91.30 ± 33.73	36 (97.3%)	74.30 ± 22.70	69 (87.3%)
Calcium (mg)	957.98 ± 401.20	20 (39.2%)	789.87 ± 250.12	17 (18.9%)	866.35 ± 339.90	12 (32.4%)	799.72 ± 331.66	21 (26.6%)
Magnesium (mg)	274.15 ± 109.22	12 (23.5%)	215.90 ± 62.47	2 (2.2%)	251.41 ± 99.67	6 (16.2%)	216.47 ± 69.83	4 (5.1%)
Iron (mg)	14.83 ± 6.20	18 (35.3%)	11.53 ± 3.56	17 (18.9%)	13.15 ± 4.20	11 (29.7%)	12.17 ± 3.61	13 (16.5%)
Sodium (mg)	2423.41 ± 991.93 **	13 (25.5%)	1927.49 ± 698.91 **	4 (4.4%)	1760.35 ± 647.44	2 (5.4%)	1746.05 ± 753.45	6 (7.6%)
Potassium (mg)	2838.09 ± 1057.49	11 (21.6%)	2378.20 ± 796.82	9 (10.0%)	2641.40 ± 1005.22	7 (18.9%)	2425.72 ± 897.54	9 (11.4%)
Phosphorous (mg)	1438.33 ± 516.07	50 (98.0%)	1167.55 ± 318.25	84 (93.3%)	1235.28 ± 493.50	35 (94.6%)	1129.95 ± 410.08	71 (89.9%)
Iodine (µg)	49.32 ± 37.20	1 (2.0%)	51.55 ± 38.34	2 (2.2%)	54.97 ± 28.00	0	55.52 ± 46.44	1 (1.3%)
Vitamin A (µg)	1677.96 ± 1087.34	44 (86.3%)	1674.59 ± 1004.53	73 (81.1%)	1830.12 ± 1014.57	32 (86.5%)	1857.02 ± 1026.92	71 (89.9%)
Vitamin C (mg)	148.26 ± 85.82	40 (78.4%)	136.54 ± 78.27	63 (70.0%)	153.96 ± 65.60	32 (86.5%)	155.17 ± 81.40	68 (86.1%)
Vitamin D (µg)	9.58 ± 8.56	28 (54.9%)	6.88 ± 8.57	38 (42.2%)	10.30 ± 9.93	22 (59.5%)	7.64 ± 5.91	45 (57.0%)
Vitamin E (mg)	9.24 ± 8.79	8 (15.7%)	6.16 ± 2.78	0	6.61 ± 4.59	2 (5.4%)	6.35 ± 3.83	3 (3.8%)
Vitamin B1 (mg)	2.08 ± 1.14	44 (86.3%)	1.73 ± 2.33	66 (73.3%)	1.87 ± 0.72	31 (83.8%)	1.67 ± 0.64	59 (74.7%)
Vitamin B2 (mg)	1.99 ± 0.95	43 (84.3%)	1.62 ± 1.59	62 (68.9%)	1.67 ± 0.73	24 (64.9%)	1.50 ± 0.66	46 (58.2%)
Vitamin B6 (mg)	2.39 ± 1.34	40 (78.4%)	1.88 ± 2.23	59 (65.6%)	2.10 ± 0.95	30 (81.1%)	1.76 ± 0.74	52 (65.8%)
Vitamin B8 (µg)	1.96 ± 10.18	1 (2.0%)	0	0	1.23 ± 3.99	0	0	0
Vitamin B9 (µg)	99.59 ± 36.36	51 (100%)	78.44 ± 29.43	90 (100%)	94.39 ± 31.14	37 (100%)	82.75 ± 32.12	79 (100%)
Vitamin B12 (µg)	6.97 ± 5.34	47 (92.2%)	5.10 ± 5.52	73 (81.1%)	6.02 ± 5.18	32 (86.5%)	6.81 ± 9.67	62 (78.5%)
SFAs (g/day)	27.54 ± 11.76	51 (100%)	25.12 ± 9.89	89 (98.9%)	26.84 ± 11.55	36 (97.3%)	25.66 ± 11.16	79 (100%)
MUFAs (g/day)	33.04 ± 11.20	50 (98.0%)	30.53 ± 12.01	87 (96.7%)	31.24 ± 12.88	36 (97.3%)	29.48 ± 12.36	76 (96.2%)
PUFAs (g/day)	9.86 ± 5.74	17 (33.3%)	7.61 ± 3.50	20 (22.2%)	8.88 ± 4.94	12 (32.4%)	7.51 ± 3.78	16 (20.3%)
Total cholesterol (mg/day)	466.75 ± 200.82	40 (78.4%)	367.69 ± 148.22	54 (60.0%)	434.92 ± 188.84	27 (73.0%)	422.07 ± 188.73	59 (74.7%)
Fibre (g/day)	13.41 ± 6.04	50 (98.0%)	12.96 ± 5.36	89 (98.9%)	14.77 ± 6.95	35 (94.6%)	13.45 ± 5.78	74 (93.7%)
Alcohol (g/day)	4.81 ± 11.43 **	2 (3.9%)	3.13 ± 7.94 **	1 (1.1%)	0.29 ± 1.26	0	0.44 ± 1.89	0

Data are mean values ± SDs and percentages; DRIs, Dietary reference intakes; SFAs, Saturated fatty acids; MUFAs, Monounsaturated fatty acids, PUFAs, Polyunsaturated fatty acids; ** Student’s *t*-test according to Christian or Muslim religion; *p* < 0.001.

**Table 4:** Correlations (R) between energy, food group serving intake, body mass index, blood pressure, waist-to-hip ratio and body adiposity index measured among Christian males and females.

Variables	Males (*n* = 51)					Females (*n* = 90)				
	BMI (kg/m^2^)	SBP (mmHg)	DBP (mmHg)	WHR	BAI (cm/m^1.5–18^)	BMI (kg/m^2^)	SBP (mmHg)	DBP (mmHg)	WHR	BAI (cm/m^1.5–18^)
Energy (kcal)	0.348 **	0.325	0.197	0.285	0.341 **	0.278 *	0.279	0.185	0.242	0.242
Bread, grains, pasta	0.182	0.289	0.333 *	0.186	0.279	0.179	0.264	0.204	0.165	0.249
Milk and derivatives	0.184	0.124	0.097	0.219	0.272	0.002	0.101	0.068	0.134	0.231
Greens and vegetables	−0.169	−0.271	−0.166	−0.130	−0.299	−0.154	−0.251	−0.149	−0.124	−0.267
Fruit	−0.174	−0.232	−0.129	−0.146	−0.257	−0.165	−0.220	−0.110	−0.124	0.238
Legumes	−0.185	−0.163	−0.146	−0.103	−0.241	−0.167	−0.153	−0.138	−0.100	−0.235
Fish and seafood	−0.267	−0.259	−0.166	−0.109	−0.221	−0.251	−0.244	−0.123	−0.101	−0.216
Lean meats	−0.254	−0.212	−0.155	−0.250 *	−0.301	−0.231	−0.202	−0.149	−0.110	−0.277
Eggs	0.261	0.204	0.188	0.152	0.287	0.241	0.189	0.174	0.141	0.269
Sausages, fatty meats	0.310	0.328	0.187	0.265	0.332 *	0.287	0.287	0.162	0.226	0.299 *
Water	0.104	0.054	0.097	0.067	0.176	0.081	0.025	0.008	0.067	0.106
Sweets, snacks, soft drinks	0.309 **	0.288	0.210	0.274	0.333	0.296 *	0.258	0.199	0.249	0.287

Notes. BMI = Body mass index; SBP = Systolic blood pressure; DBP = Diastolic blood pressure; WHR = Waist-to-hip ratio; BAI = Body adiposity index. * *p* < 0.05; ** *p* < 0.01.

**Table 5:** Correlations (R) between energy, food group serving intake, body mass index, blood pressure, waist-to-hip ratio and body adiposity index measured among Muslim males and females.

Variables	Males (*n* = 37)					Females (*n* = 79)				
	BMI (kg/m^2^)	SBP (mmHg)	DBP (mmHg)	WHR	BAI (cm/m^1.5–18^)	BMI (kg/m^2^)	SBP (mmHg)	DBP (mmHg)	WHR	BAI (cm/m^1.5–18^)
Energy (kcal)	0.327 *	0.287	0.183	0.343 *	0.321	0.298 *	0.274	0.169	0.299 *	0.297
Bread, grains, pasta	0.187	0.212	0.199	0.177	0.251	0.164	0.189	0.174	0.141	0.197
Milk and derivatives	0.154	0.133	0.077	0.187	0.273	0.137	0.110	0.041	0.157	0.224
Greens and vegetables	−0.333 *	−0.273	−0.168	−0.117	−0.298 *	−0.219	−0.229	−0.082	−0.110	−0.231
Fruit	−0.173	−0.201	−0.132 *	−0.134	−0.237	−0.169	−0.189	−0.021	−0.109	−0.224
Legumes	−0.181	−0.291 **	−0.131	−0.110	−0.219	−0.157	−0.182	−0.114	−0.089	−0.194
Fish and seafood	−0.256	−0.299 *	−0.184 **	−0.112	−0.197	−0.259	−0.198	−0.087	−0.071	−0.184
Lean meats	−0.288 **	−0.232 *	−0.140	−0.246 *	−0.294 **	−0.127	−0.154	−0.121	−0.135	−0.288
Eggs	0.258	0.201	0.181	0.147	0.271	0.239	0.193	0.176	0.131	0.263
Sausages, fatty meats	0.312	0.315	0.174	0.242	0.357 *	0.297 *	0.298 *	0.151	0.237	0.288 *
Water	0.094	0.056	0.101	0.061	0.169	0.096	0.042	0.073	0.058	0.157
Sweets, snacks, soft drinks	0.298 **	0.261	0.201	0.256	0.327 **	0.289 *	0.250	0.179	0.245	0.301 *

Notes. BMI = Body mass Index; SBP = Systolic blood pressure; DBP = Diastolic blood pressure; WHR = Waist-to-hip ratio; BAI = Body adiposity index. * *p* < 0.05; ** *p* < 0.01.

**Table 6:** Belonging to the Christian or Muslim religion and its associations with variables related to cardiovascular risk.

Variables	*n*	%	OR	95% CI	OR ^a^	95% CI
**Normal weight**						
Yes	100	53.2	1			
No	41	59.4	1.29	0.74; 2.25	1.39	0.74; 2.61
**WHR**						
Normal	96	54.2	1			
Elevated	45	56.2	1.09	0.64; 1.85	0.91	0.53; 1.55
**Visceral fat indicator**						
Normal	128	53.1	1			
Elevated	13	81.2	3.83 *	1.06; 13.77	0.27 *	0.07; 0.97
**Blood pressure**						
Normal	71	55.0	1			
Elevated	70	54.7	0.99	0.60; 1.61	1.10	0.65; 1.88
**Fat mass (%)**						
Normal	70	55.1	1			
Elevated	71	54.6	0.98	0.60; 1.60	1.01	0.62; 1.65
**Cigarette consumption**						
Yes	39	68.4	1			
No	102	51.0	2.08 *	1.15; 3.88	1.93	0.98; 3.82
**Consumption of alcoholic beverages**						
Yes	112	77.2	1			
No	29	25.9	9.71 **	5.47; 17.24	7.88 **	4.27; 14.54
**Practise a sport**						
Yes	76	58.0	1			
No	65	51.6	1.30	0.79; 2.12	1.12	0.63; 2.02

Christians were taken as reference. Data are presented as odds ratios (OR) with 95% confidence intervals (CIs) using a logistic regression model. OR a adjusted for sex and parents’ education levels. * *p* < 0.05; ** *p* < 0.001.

## Abbreviations

The following abbreviations are used in this manuscript:

BMI	body mass index
SBP	systolic blood pressure
BAI	body adiposity index
CNCDs	chronic noncommunicable diseases
DRIs	dietary reference intakes
TEM	technical measurement error
BP	blood pressure
ESH	European Society of Hypertension
ESC	European Society of Cardiology
WHR	waist-to-hip ratio
LDL-C	low-density lipoprotein cholesterol
ABSI	a body shape index
SFAs	saturated fatty acids
MUFAs	monounsaturated fatty acids
PUFAs	polyunsaturated fatty acids
SD	standard deviation
OR	odd ratio
CI	confidence interval
